# Facile fabrication of mesoporous silica micro-jets with multi-functionalities†
†Electronic supplementary information (ESI) available: Detailed BET experiments, videos and supplementary data. See DOI: 10.1039/c7nr04527a


**DOI:** 10.1039/c7nr04527a

**Published:** 2017-08-17

**Authors:** D. Vilela, A. C. Hortelao, R. Balderas-Xicohténcatl, M. Hirscher, K. Hahn, X. Ma, S. Sánchez

**Affiliations:** a Max Planck Institute for Intelligent Systems , Heisenbergstr. 3 , 70569 Stuttgart , Germany . Email: ssanchez@ibecbarcelona.eu ; Email: sanchez@is.mpg.de; b Institute for Bioengineering of Catalonia (IBEC) , The Barcelona Institute of Science and Technology , Baldiri Reixac 10-12 , 08028 Barcelona , Spain; c State Key Laboratory of Advanced Welding and Joining , Harbin Institute of Technology (Shenzhen) , Shenzhen 518055 , China . Email: maxing@hit.edu.cn; d Key Laboratory of Micro-systems and Micro-structures Manufacturing of Ministry of Education , Harbin Institute of Technology , Harbin 150001 , China; e Institució Catalana de Recerca i Estudis Avançats (ICREA) , Psg. Lluís Companys , 23 , 08010 Barcelona , Spain

## Abstract

Self-propelled micro/nano-devices have been proved as powerful tools in various applications given their capability of both autonomous motion and on-demand task fulfilment. Tubular micro-jets stand out as an important member in the family of self-propelled micro/nano-devices and are widely explored with respect to their fabrication and functionalization. A few methods are currently available for the fabrication of tubular micro-jets, nevertheless there is still a demand to explore the fabrication of tubular micro-jets made of versatile materials and with the capability of multi-functionalization. Here, we present a facile strategy for the fabrication of mesoporous silica micro-jets (MSMJs) for tubular micromotors which can carry out multiple tasks depending on their functionalities. The synthesis of MSMJs does not require the use of any equipment, making it facile and cost-effective for future practical use. The MSMJs can be modified inside, outside or both with different kinds of metal nanoparticles, which provide these micromotors with a possibility of additional properties, such as the anti-bacterial effect by silver nanoparticles, or biochemical sensing based on surface enhanced Raman scattering (SERS) by gold nanoparticles. Because of the high porosity, high surface area and also the easy surface chemistry process, the MSMJs can be employed for the efficient removal of heavy metals in contaminated water, as well as for the controlled and active drug delivery, as two proof-of-concept examples of environmental and biomedical applications, respectively. Therefore, taking into account the new, simple and cheap method of fabrication, highly porous structure, and multiple functionalities, the mesoporous silica based micro-jets can serve as efficient tools for desired applications.

## Abstract 2

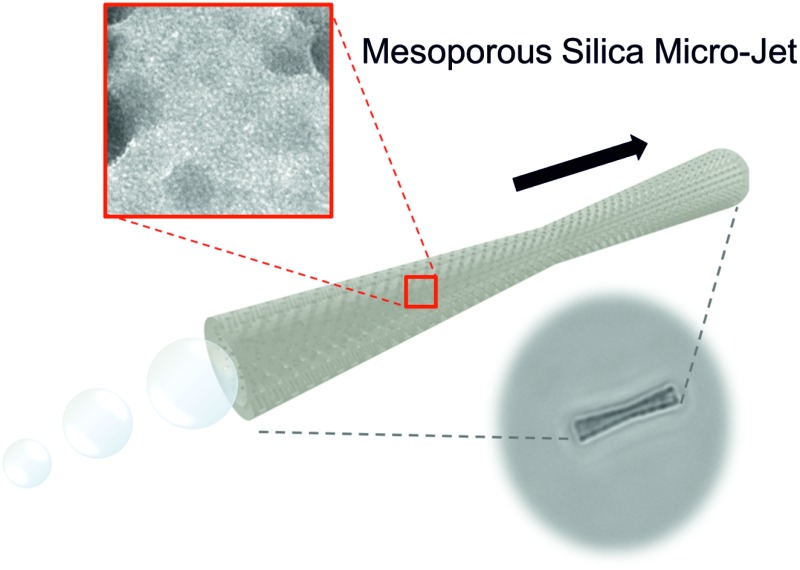
Facile strategy for the fabrication of mesoporous silica micro-jets (MSMJs) as novel structure of tubular micromotors which can serve as a common platform able to carry out different tasks *via* surface functionalities.

## Introduction

The field of micro/nano-motors has experienced a fast growth during the past decade, which has attracted increasing scientific interest and consequently various techniques have been developed for the fabrication of different micro/nano-motors.^1^–^3^ In particular, tubular micro-jets have one of the most promising structures and several methods have been designed for their preparation. Rolling-up of thin films and electrochemical deposition techniques are the most common strategies used to prepare the three-dimensional (3-D) tubular micro-structure to construct micro-jets. On the one hand, the rolling-up method, although clean room based, time and energy consuming, has the advantage that the size and the components of the tubular structure can be readily tuned by varying the pattern size of the photoresist during the photolithography process and by using different materials during the e-beam deposition, respectively.^4^,^5^ Researchers have also developed some other rolling-up methods to prepare tubular structures for the fabrication of tubular-jets which are clean room-free.^6^–^9^ On the other hand, the electrochemical deposition technique uses membrane templates (*e.g.* Al_2_O_3_ or polycarbonate) which contain micropores (*e.g.* 2 × 20 μm and 5 × 20 μm, *D* × *L*). To fabricate micro/nanomotors by the electrochemical deposition process, various materials, including conductive polymers or metals, are deposited onto the inner surface of the micro-pores from membranes.^10^,^11^ Another method uses these membranes as templates to prepare tubular structures such as the self-assembly technique. This approach is based on the self-assembly of organic molecules, such as polymers and proteins, inside the micro-pores.^12^,^13^ Regarding the template based method, we recently used silver nanowires as a template to prepare ultra-small silica nano-jets.^14^ Even though quite a few methods are available in the current pool, efforts on the exploration of new materials to construct tubular micro-jets will endow new functionalities and capabilities to these self-propelled devices.

**Scheme 1:**
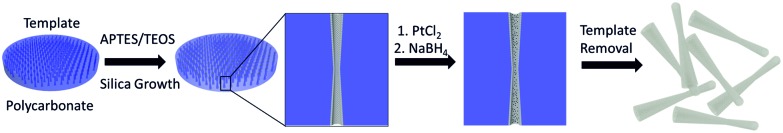
Schematic illustration of the fabrication of mesoporous silica tube based micro-jets (MSMJs).

In order to make use of these self-propelled tubular-jets, researchers have integrated different functionalities within these micro-jets leading to various active micro/nano-devices capable of fulfilling on-demand tasks in aqueous solutions,^1^,^15^,^16^ including applications in the fields of biomedical sensing, environmental protection, *etc*.^17^,^18^ For instance, tubular jets have been used as vehicles for biomedical purpose,^19^ such as the drilling into cancer cells,^20^ and controlled and targeted anti-cancer drug (Doxorubicin) delivery towards cancer cells *in vitro*.^13^ Tubular micro-jets have also been used as “on-the-fly” sensors and biosensors recognizing inorganic (toxic metals) molecules,^21^ and isolating biomolecules on-demand, such as specific proteins and DNA sequences.^22^,^23^ Furthermore, tubular micro-jets were utilized to address environmental problems,^24^ such as organic pollutants degradation^25^ or heavy metal removal^11^ from contaminated water where the surface properties of the tubular micro-jets play a critical role. Therefore, surface modification on the tubular structure of these micro-jets, as well as their cargo loading efficiency become rather necessary to achieve the desired functionality. Moreover, most of the current tubular micro-jets can only accomplish a single task, since usually the material with which these micro-jets are made of and their fabrication process are designed for single application, which cannot satisfy the requirement of multi-functionality for future intelligent self-propelled systems. Therefore, novel methods for the preparation of tubular jets comprising new materials, which can provide a versatile platform and new capabilities that can be readily adapted for different tasks *via* easy surface functionalization, are in demand. Hereby, we present a facile strategy for the fabrication of mesoporous silica micro-jets (MSMJs) as a novel structure of tubular micromotors which can serve as a common platform able to carry out different tasks *via* surface functionalities. The MSMJs are based on biconical tubes made of mesoporous silica containing platinum nanoparticles (PtNPs) in their inner surface (Scheme 1). The PtNPs decompose hydrogen peroxide (H_2_O_2_) into water and oxygen, generating oxygen microbubbles ejected from one side of micro-jets and providing the driving force for the self-propulsion. Mesoporous silica is advantageous for the construction of the tubular structure of micro-jets, in virtue of its extremely large surface area, large pore volume, transparency and ease of surface chemical modifications^26^ which can endow the tubular micro-jets with a strong capability of cargo loading *via* physical adsorption on the large surface or encapsulation into the nanopores’ cavities.^27^

## Methods

### Materials and instruments

Tethaethylorthosilicate (TEOS, 99%), cetyltrimethylammonium bromide (CTAB, 99%), 3-aminopropyltriethoxysilane (APTES, 99%), triethanolamine (TEOA, 98%), (3-mercaptopropyl) trimethoxysilane (MPTMS, 99%), sodium dodecyl sulfate (SDS), silver nitrate, sodium borohydride, polyvinylpyrrolidone (PVP), gold(iii) chloride trihydrate, sodium citrate, lead nitrate, nitrate acid, Rhodamine B, hydrochloric acid, potassium dihydrogen phosphate, sodium acetate and acetic acid were purchased from Sigma-Aldrich (Germany). Hydrogen peroxide (H_2_O_2_, 30%), methylene chloride and ethanol were purchased from Merck (Germany). Ultrapure water (Millipore Corporation, USA) was used for the preparation of all aqueous solutions.

The synthesis of mesoporous silica micro-jets (MSMJs) was carried out using a simple heater with an integrated magnetic stirrer and temperature control thermometer (IKA). A spectrophotometer Specord 50/plus (Analytik Jena, Germany) was employed to characterize the synthesized AgNPs and AuNPs. An inverted optical microscope (Leica DMI3000B), coupled with 10×, 20×, 40× and 63× objectives, along with a Leica digital camera DFC3000G with LAS V4.5 software, were used for making images and recording movies. Furthermore, an inverted microscope (Leica DMi8) coupled with a filter cube for rhodamine and 10×, 20×, 40× and 63× objectives was used to acquire fluorescence images. High-angle annular dark-field (HAADF) images and STEM-EELS/EDX-images were recorded in a JEOL ARM200CF scanning transmission electron microscope equipped with a cold field emission source, a DCOR probe corrector (CEOS GmbH), a 100 mm^2^ JEOL Centurio EDX detector and a Gatan GIF ERS electron energy-loss spectrometer. Square wave stripping voltammetry (AUT50101, Metrohm Autolab B.V.) was employed as the analytical technique for the detection of lead (Pb). Nova 1.11, Origin Pro 9.0, Microsoft Excel 2013 and ImageJ were used for the analysis of the experimental data.

### Fabrication of the mesoporous silica tubes (MSTs)

The mesoporous silica microtubes (MSTs) were synthesized using as a template a cyclopore polycarbonate membrane, containing conical-shaped micro-pores with 2 μm maximum diameter (catalog no. 7060-2511; Whatman, Maidstone, UK). CTAB (12.5 mg) and TEOA (10 mg) were dissolved in 4 mL of water in a glass vial where the polycarbonate membrane was placed. The mixture was heated and when it reached 80 °C, 5 μL of APTES were added under stirring. After 30 min at constant temperature, 30 μL of a mixture of 15 and 75 μL of APTES and TEOS, respectively, were added and the reaction was kept at 80 °C for 2 more hours. The mixture solution was kept stirring for 20 minutes at room temperature. The membrane was rinsed with water and polished on both sides using a cotton swab. Afterwards, the polycarbonate membrane template was dissolved in CH_2_Cl_2_ for 15 min and the free mesoporous silica motors were washed 2 times in CH_2_Cl_2_, 2 times in EtOH and 2 times in H_2_O. The MSTs were stored in water at room temperature. Scheme 1 describes the detailed fabrication of the self-propelled mesoporous silica micro-jets (MSMJs).

### Fabrication of the mesoporous silica micro-jets (MSMJs)

The polished polycarbonate membrane with the MSTs inside its pores was incubated with a solution (H_2_O : EtOH = 1 : 1) saturated with PtCl_2_ salt for 4 h. Then, after rinsing it, the membrane was submerged in a NaBH_4_ (20 mM) solution for 30 min, obtaining platinum nanoparticles (PtNPs) on the inner layer of the MSTs. Afterwards, the polycarbonate membrane template was dissolved in CH_2_Cl_2_ over 15 min and the free MSMJs were washed 2 times in CH_2_Cl_2_, 2 times in EtOH and 2 times in H_2_O. The MSMJs were stored in water at room temperature.

### Thiol functionalization of the mesoporous silica tubes

MSTs were suspended in methanol (1 mL) containing 10 μL of (3-mercaptopropyl) trimethoxysilane. The reaction was kept at room temperature for 48 h under strong stirring. Afterwards the SH-MSTs were washed with EtOH 3 times. The SH-MSTs were stored in EtOH at room temperature.

### Fabrication of gold nanoparticles

Gold nanoparticles (AuNPs) were fabricated by reduction of gold(iii) chloride trihydrate with sodium citrate as a reducer and stabilizer.^28^ Prior to the preparation of AuNPs, all necessary glassware was cleaned using freshly prepared aqua Regia, rinsed thoroughly in water, and dried in air for 24 hours. 100 mL of 1 mM of AuCl^4–^ solution was boiled in a round bottom flask with a magnet and integrated in a reflux system. Then, 10 mL of 38.8 mM NaCit was added and the solution was boiled for 20 min turning red in color. Finally, the solution was left under stirring without heating for 1 hour until it reached room temperature. The AuNP colloidal solution was stored at room temperature in the dark. The average diameter of the synthesized AuNPs was 12 ± 3 nm, quantified by *n* = 20.

### Fabrication of silver nanoparticles

Silver nanoparticles (AgNPs) were fabricated by reduction of silver nitrate with NaBH_4_ in the presence of PVP as a stabilizer.^29^ Prior to the preparation of AgNPs, all necessary glassware was washed using freshly prepared aqua Regia, rinsed thoroughly in water, and dried in air for 24 hours. An Erlenmeyer flask with 10 mL of 0.02 M NaBH_4_ and 10 mL of 0.375 M PVP was placed in an ice bath with constant stirring for 20 min. Then, 10 mL of 0.02 M AgNO_3_ solution was added dropwise into the solution until the solution became vivid dark yellow. The AgNP colloidal solution was washed three times in ethanol by centrifugation at 13 000 rpm for 10 min. AgNPs were characterized by carrying out UV-Visible spectroscopy and TEM. The average diameter of the synthesized AgNPs was 23 ± 6 nm, quantified by *n* = 20.

### Fabrication of AgNP based MST and AuNP based MST

AgNP based MST (AgNP-MST) and AuNP based MST (AuNP-MST) were prepared by incubating 450 μL of the SH-MST stock solution with 50 μL of AgNPs and AuNPs under stirring for 1 hour, respectively. After incubation, each sample was cleaned 3 times with ETOH and twice in H_2_O. Then, AgNP-MST and AuNP-MST were stored in water at room temperature.

### Removal of lead using SH-MSMJs

Lead contaminated solutions (1 ppm) were in the presence of SH-MSMJs (*n* = 3) and MSMJs for 30 min (total volume 1 mL). Afterwards the samples were centrifuged and the supernatants were diluted 10 times in 0.02 M acetate buffers (pH 4.8), respectively. The amount of lead in the solutions was measured by electrochemistry.

### Quantification of heavy metals

Lead was measured by square wave voltammetry using a mercury coated glassy carbon electrode. The mercury film was pre-plated at the beginning from a non-deaerated, stirred 80 mg L^–1^ mercury solution (in 0.02 M HCl), by holding the carbon strip electrode at –1.15 V for 15 min. The potential was then switched to –0.20 V for a 2 min cleaning period. The subsequent cycles involved the lead deposition (3 min of pre-concentration at –1.15 V) and stripping steps with the potential scanned and stopped at –0.2 V. 0.02 M acetate buffer (pH 4.8) was used as an electrolyte.^30^

For the calibration curve of lead, the range of concentrations of lead which followed a linear correlation (*r* = 0.990) was between 0.01–0.1 ppm. Afterwards, the amount of lead was measured before (0.1 ppm) and after the treatment with MSJs and SH-MSJs.

### Cargo loading and release of Rhodamine B into the mesoporous silica microtubes (MSTs)

200 μL of microtubes were collected and centrifuged. Then, the supernatant was removed and the microtubes were re-suspended in 1 mL of a solution of 0.5 mg mL^–1^ of Rhodamine B in a mixture of isopropanol and H_2_O (10 : 2). The mixture was shaken end-to-end for 24 h. Later, the microtubes were washed 4 times with a mixture of isopropanol and H_2_O, by centrifuging, carefully removing the supernatant and re-suspending it in a fresh mixture.

Time-dependent release of cargo: After washing, the MSTs were re-suspended in Milli-Q water and fluorescence images were acquired. To analyse the time-dependent release of Rhodamine B from the MSTs, images were acquired at time 0, thereafter every hour for 8 consecutive hours and after 24 h maintaining the same microscope parameters for all time points.

### Surface area and pore size analysis

**Fig. 1:**
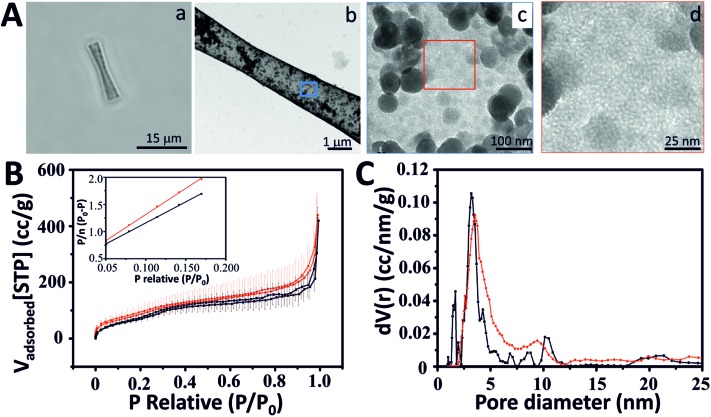
Characterization of mesoporous silica microtubes (MSTs). (A) Mesoporous silica microtube (MST): (a) optical image of a MST, (b) TEM image of a MST, TEM images of the MST surface at low (c) and (d) high magnification. (B) Ar (black) and N_2_ (red) adsorption isotherms at the corresponding condensation temperature (for error bars see the ESI†) (inset: argon (black) and nitrogen (red) BET plot using a relative pressure range (0.05–0.2)). (C) Pore size distribution calculated using Ar and N_2_ isotherms and NLDFT.

A fully automated Sierverts’ apparatus (Quatachrome Autosorb iQ^2^) was used for the gas adsorption experiments. The experiments were carried out for Ar and N_2_ at the respective condensation temperature controlled by a cryocooler with a temperature stability <0.05 K. BET analysis was performed in a relative pressure range (0.05–0.2) since pore condensation occurs close to *P*/*P*_0_ = 0.3 (Fig. 1B) (for details, see the ESI†).

### TEM and STEM images

For all the TEM and STEM characterization, 5 μL of each sample were deposited on a copper grid (3 mm, PLANO GmbH) and left for drying overnight at room temperature before the observations and image capture.

## Results and discussion

**Fig. 2:**
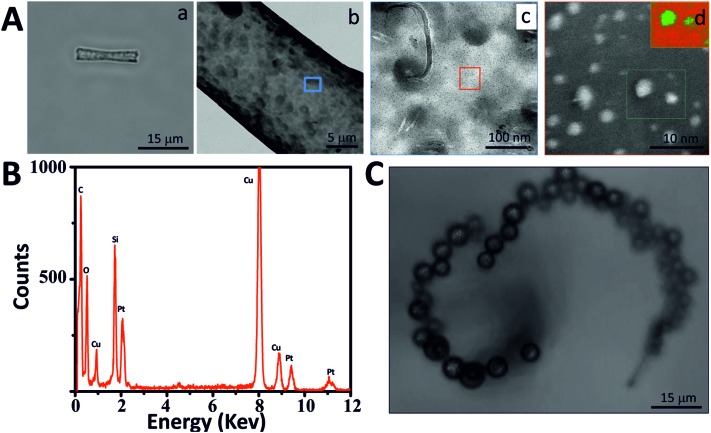
Characterization of mesoporous silica micro-jets (MSMJs). (A) MSMJ with PtNPs in the inner surface: (a) optical image of a MSMJ, (b) TEM image of a MSMJ, (c) TEM image of the MSMJ surface and (d) STEM image of PtNPs in MSMJ (inset: EELS mapping. PtNPs (green) and silica (red)). (B) EDX analysis of MSMJs. (C) Picture of a MSMJ swimming in aqueous solution (0.5% SDS and 1.5% H_2_O_2_).

MSTs and MSMJs were characterized by transmission electron microscopy (TEM), scanning transmission electron microscopy (STEM), energy dispersive X-ray spectroscopy (EDX) and Brunauer–Emmett–Teller (BET) surface area analysis (Fig. 1 and 2). Fig. 1 displays the characterization of the MST structure. An optical image of MSTs reveals their biconical shape, their length which was found to be 17 ± 1 μm (average ± standard error of the mean, *n* = 20) and their transparent properties (Fig. 1Aa). Fig. 1Ab–Ad illustrate the characterization of the surface of the MSTs using TEM. As is observed in Fig. 1Ab and Ac, the MST surface is not homogeneous, small silica agglomerations were noted which show how silica grew inside the template pores. Finally, Fig. 1Ad shows the mesoscale pores on the silica tubes, which provide the micro-jets with a high surface area. The physical properties of the MSTs were also evaluated using high-resolution adsorption experiments for N_2_ and Ar at the respective condensation temperature.^31^ MSTs exhibit type-IV isotherms where capillary condensation is noted by the change in the isotherm between the relative pressures (*P*/*P*_0_) 0.25 and 0.75, confirming the presence of the mesoporous structure (Fig. 1B). The N_2_ and Ar BET specific area of MSTs was found to be 309 and 235 m^2^ g^–1^, respectively (Table S1 in ESI†). The estimated average pore diameter was found to be 3.8 nm (Fig. 1C).

Fig. 2 illustrates the characterization of the MSMJs. As was previously described, MSMJs are MSTs which have in their inner surface PtNPs for their self-propulsion in the presence of H_2_O_2_. Fig. 2Aa displays a MSMJ which shows a darker color due to the presence of PtNPs inside without losing its transparent properties. The rough and non-homogenous surface of the jets is observed in Fig. 2Ab. Fig. 2Ac and Ad show the PtNPs synthesized on the inner surface of a MSMJ. The size of the synthesized PtNPs in the MSMJs was measured using STEM revealing a large polydispersity with diameters between 1–4 μm. In order to prove the presence of PtNPs, EELS mapping (inset, Fig. 2Ad) and EDX analysis (Fig. 2B) were carried out, confirming the presence of platinum in the silica structure. Additionally, the swimming of the MSMJs was studied observing velocities up to 320 μm s^–1^ (∼15 body lengths per s) in a low concentration of peroxide (1.5%) (Fig. 2C).

**Fig. 3:**
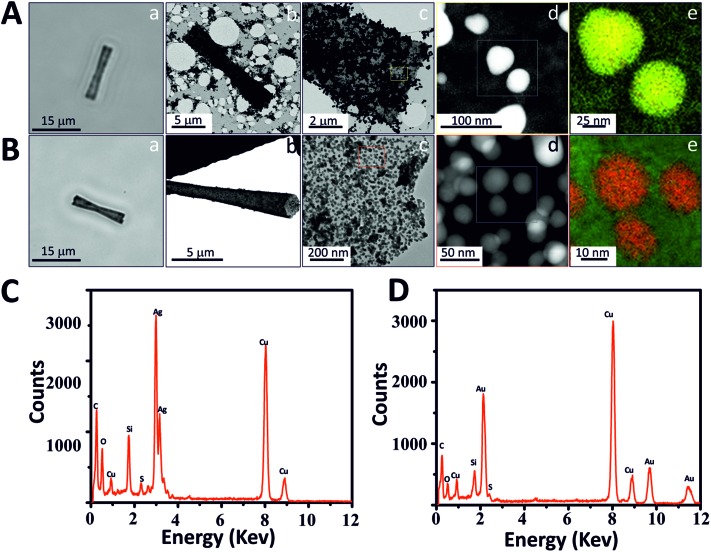
Modification of the MST with AuNPs and AgNPs. (A) AgNP mesoporous silica tube (AgNP-MST): (a) optical image of a AgNP-MST, (b) TEM image of a AgNP-MST, (c) TEM image of the AgNP-MST surface, (d) STEM-EELS image of the AgNPs on MST and (e) element mapping (AgNPs (yellow) and silica (green)). (B) AuNP mesoporous silica tube (AuNP-MST): (a) optical image of AuNP-MST, (b) TEM image of a AuNP-MST, (c) TEM image of AuNP-MST surface, (d) STEM-EELS image of the AuNPs on MST and (e) element mapping (AuNPs (red) and silica (green)). (C) EDX analysis of AgNP-MST. (D) EDX analysis of AuNP-MST.

We explored different possible applications of these new MSMJs. First, we modified the mesoporous silica surface with different types of metal nanoparticles (MNPs) which provide special physical and chemical properties to MSMJs. To incorporate MNPs onto MSTs, the surface of MSTs has been modified to obtain thiol groups due to their high affinity to MNPs. Gold nanoparticles (AuNPs) and silver nanoparticles (AgNPs) were selected as examples because of their special properties and related wide applications in multiple fields. For instance, AuNPs are able to attach biomolecules increasing the active area of the MSMJs and also have mimicked glucose oxidase activity^32^–^35^ and AgNPs have already been proved as potent bactericidal agents for water decontamination.^36^–^39^ First, to obtain MNP based MST, AgNPs and AuNPs were synthesized, respectively, (see Methods and Fig. S2†) using common synthesis methods.^28^,^29^ Then, they were characterized using UV-vis spectroscopy and TEM (Fig. S2†) observing that AuNPs present lower polydispersity and higher stability than AgNPs. Afterwards, previously synthesized MSTs were modified to generate SH-tubes and thereafter SH-MSTs were incubated with the synthesized MNPs, respectively. To demonstrate the presence of AgNPs and AuNPs on MSTs, the MNP based MSTs were characterized (Fig. 3). Fig. 3A and C show the characterization of the AgNP based MSTs (AgNP-MSTs) and Fig. 3B and D show the characterization of the AuNP based MSTs (AuNP-MSTs). As is shown, AgNP-MSTs (Fig. 3Aa) and AuNP-MSTs (Fig. 3Ba) turn darker than MSTs since the light cannot go through the MNPs giving a hint of the heavily-covered surface of the MSTs. This fact was confirmed in Fig. 3Ab and Ac where the AgNP-MST image by TEM reveals the high concentration of AgNPs agglomerated on the MST surface and how they are distributed in clusters due to the low stability of AgNPs. However, AuNPs showed a good distribution of AuNPs along the MST surface (Fig. 3Bb) being dispersed without forming clusters due to the high stability of the AuNPs (Fig. 3Bc). As was previously observed in the TEM images of their colloidal solutions, STEM images also showed that AgNPs (Fig. 3Ad) are bigger and less polydisperse than AuNPs (Fig. 3Bd) on the silica surface. The presence of the MNPs on the MST was proved by the mapping of the elements using STEM-EELS which displayed the AgNPs in yellow color (Fig. 3Ae) and AuNPs in red color (Fig. 3Be) on silica (green color) and by EDX analysis which displayed the peaks of energy corresponding to Ag (Fig. 3C) and Au (Fig. 3D). Thus, we demonstrated the easy modification capabilities of MSMJs by the attachment of different nanostructures on their surface.

**Fig. 4:**
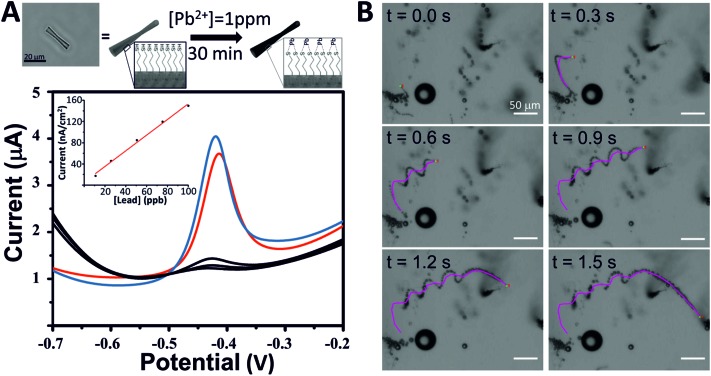
Removal of lead in water using MSMJs decorated with a thiol group. (A) Scheme of interactions between thiol (–SH) group with lead (Pb) as a model of heavy metal (above). Square wave voltammetry of the lead concentration in the absence of MSMJs (blue), after 30 min of treatment with MSMJs without thiol groups (red) and after 30 min of treatment with MSMJs decorated with thiol groups (black) (inset: represent the calibration curve for lead by square wave voltammetry) (below). (B) MSMJ swimming during the lead removal after peroxide addition (1.5% H_2_O_2_, 0.5% SDS).

As proof of the concept of these micro-jets as new tools for environmental applications, we have used them for the decontamination of heavy metals from polluted water. We selected lead (Pb) as a heavy metal model because of the high affinity of lead ions (Pb^2+^) to sulfur atoms since thiol groups may act as chelating agents of heavy metals.^40^ Taking advantage of the interactions between lead and thiols, SH-MSMJs were used to eliminate lead from water (Fig. 4A, above). Fig. 4A also shows the results corresponding to lead capturing after 30 min of treatment with a defined amount of SH-MSMJs (total volume 1 mL, 4 × 10^5^ tubes per mL). As is observed, SH-MSMJs were able to remove more than 95% of lead from 1 ppm lead contaminated water. The experiment was repeated 3 times obtaining the values of lead concentration lower than the limit of detection of the instrument (LOD = 5 ppb) as shown in the black voltammogram in Fig. 4A. Furthermore, MSMJs without thiol functionalization were also tested as a control experiment, where the removal of lead was not observed (red voltammogram, Fig. 4A). Thus, it has been proven that SH-MSMJs can be used for environmental applications such as heavy metal remediation. Fig. 4B and Video S1† display the MSMJ behavior of the MSMJs during lead removal.

**Fig. 5:**
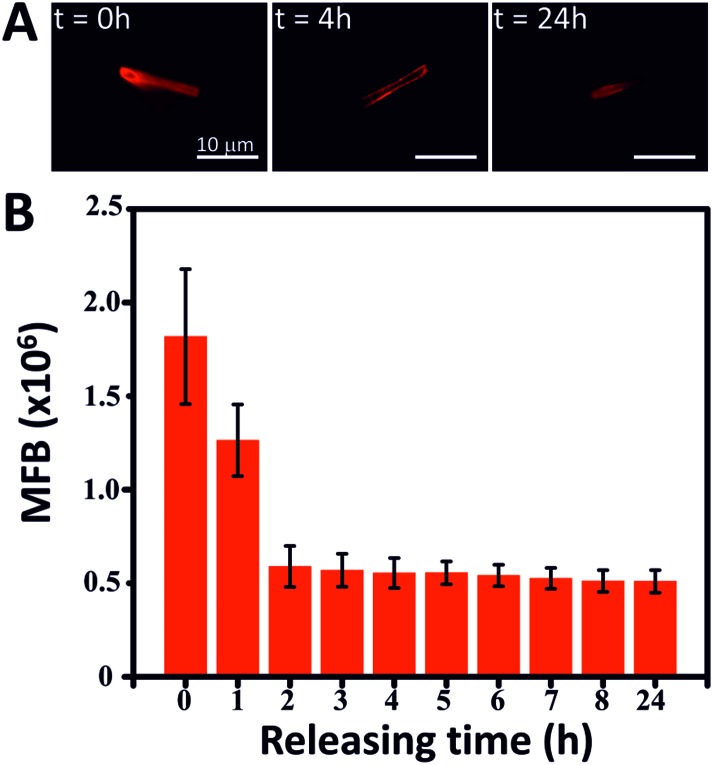
Drug delivery proof of the concept using Rhodamine B (A) pictures of the fluorescence emitted by the MST loaded with Rhodamine B (*t* = 0 h) and after 4 and 24 h of the drug release and (B) fluorescence intensity emitted by the drug in MST *vs.* releasing time (h).

Another possible application of the MSMJs is their use in the nanomedicine field as a drug delivery vehicle. As has been reported, mesoporous silica has been used as a carrier of different kinds of drugs (antitumor drugs or antibiotics among others) due to its high capacity to accommodate organic molecules in its structure and afterwards releasing them in a controlled manner.^41^–^44^ Taking advantage of the mesoporous silica structure of the MSMJs, we demonstrate their ability to carry drug molecules to a precise location and deliver them in a controlled manner. Here, Rhodamine B (Rhd B) was used as a model drug because it is an organic molecule which can emit fluorescence, being possible to correlate the intensity of the fluorescent emission with the amount of Rhd B. Therefore, the MSTs were loaded with a saturated solution of Rhd B and after several washings, the Rhd B release was followed by imaging every hour the fluorescence emission of the MST (Fig. 5). Fig. 5A shows a sharp decrease of the intensity of the fluorescence emission from the Rhd B loaded MST over time in the presence of water. This fact is confirmed in Fig. 5B where the intensity of the fluorescence emission was measured over time until 24 hours observing that Rhd B was mostly released during the first 2 hours. After that time, the intensity of the emission of the loaded MST remained almost constant. Thus, the core structure of the MSMJs has been demonstrated as a good drug carrier, being able to load model molecules on their surface and release them by diffusion, which can be controlled by tuning the motion of the MSMJs. They add a new vehicle to the library of systems for drug delivery assays and future medical applications. Furthermore, considering the development of recent studies on enzyme powered micro- and nano-motors,^45^–^50^ by replacing the inorganic catalyst (PtNP) by enzymes, such motors are capable of swimming using biocompatible and bioavailable fuels such as glucose or urea, leading to an even more promising future for biomedical purpose.

## Conclusions

Here, we have presented self-propelled tubular micro-jets made of mesoporous silica and also demonstrated their capability to develop multi-functionality. The fabrication of the mesoporous silica micro-jets (MSMJs) is carried out using a simple methodology which involves the growth of mesoporous silica on the walls of the pores of a polycarbonate membrane. The synthesis of MSMJs does not require the use of special equipment, making them facile and cost-effective for future practical use. In addition, due to the versatile surface of the MSMJs, we have shown various applications by simply attaching different nanostructures or modifying with functional groups, which provide the micro-jets with additional properties and capacities for desired applications. The MSMJs have been proven as efficient heavy metal removal tools, as well as active drug delivery vehicles, as an example of environmental and medical applications, respectively. Thus, the easily fabricated novel self-propelled micro-jets presented in this work offer a wide range of possibilities for their use in several applications in multiple fields because of the multiple functionalities on their versatile surface.

## Conflicts of interest

There are no conflicts to declare.

## Supplementary Material

Supplementary information

Supplementary movie
