# Supplementary material for: Facile fabrication of mesoporous silica micro-jets with multi-functionalities
Source: Nanoscale. 2017 Aug 17;9(37):13990–7. doi: 10.1039/c7nr04527a (PMC5708346; doi:10.1039/c7nr04527a)
Supplement: Supplementary file 1 [file NR-009-C7NR04527A-s001.pdf]

Electronic Supplementary Material (ESI) for Nanoscale.
This journal is © The Royal Society of Chemistry 2017

Facile Fabrication of Mesoporous Silica Micro-Jets with Multi-Functionalities

D. Vilela[a,b], A. C. Hortelao[a,b], R. Balderas-Xicohténcatl[a], M. Hirscher[a], K. Hahn[a], X. Ma[d,e]† and S. Sanchez[a,b,c]†

[a]*Max Planck Institute for Intelligent Systems, Heisenbergstr. 3, 70569 Stuttgart, Germany.*

[b]*Institute for Bioengineering of Catalonia (IBEC), Baldiri I Reixac 10-12, 08028 Barcelona, Spain.*

[c]*Institució Catalana de Recerca i Estudis Avançats (ICREA), Psg. Lluís Companys, 23, 08010 Barcelona, Spain*

[d]*Sate Key Laboratory of Advanced Welding and Joining, Harbin Institute of Technology (Shenzhen), Shenzhen 518055, China*

[e]*Key Laboratory of Micro-systems and Micro-structures Manufacturing of Ministry of Education, Harbin Institute of Technology, Harbin 150001, China*

†Corresponding authors: maxing@hit.edu.cn (Xing Ma); ssanchez@ibecbarcelona.eu and sanchez@is.mpg.de (Samuel Sanchez)

Surface area and pore size analysis

Videos:

Video S1. Tracking of a MSMJ in 1.5% of H_2O_2 and 0.5% of SDS during heavy metals removal.

Supplementary Figures S1-S2

Surface area and pore size analysis

Low-pressure High-resolution adsorption experiments.

A fully automated Sierverts´ apparatus (Quatachrome Autosorb iQ^2) was used for the gas adsorption experiments. The experiments were carried for Ar and N_2 at the respective condensation temperature controlled by a cryocooler with a temperature stability of < 0.05 K. The BET analysis was performed in a relative pressure range (0.05 - 0.2) since pore condensation occurs close to P/Po = 0.3 (Figure 1B). The Figure S1 show the BET plots with the respective linear fitting. The pore volume was determined according to the Gurvich rule and the average pore diameter was calculated under the assumption of a cylindrical pore geometry. The pore size distribution was calculated using NLDFT with the software ASiQwin 3.01. The calibration and analysis is described in the supporting information.

Calibration analysis of absorption measurements

The experiments were carried out using the automated operation mode NOVA (NO Void Analysis) based on separated calibration of the sample cell prior to the analysis. The calibration consists in an empty cell analysis carried out at the same temperature and using the same gas than the experiment. The adsorbed amount of gas in each data point in the isotherm is collected by dosing a known amount of adsorbate gas in the sample cell and then subtracting the necessary amount to fill the empty space in the sample cell.

$$n_{adsorbed} = n_{dosed} - n_{calibration}$$

For the error assessment, a second empty cell experiment was recorded and then the empty cell calibration was subtracted, the result is a line close to zero volume in units of absolute uptake (cc). The small variations from the ideal zero volume (Figure S1) are related to the thermal fluctuations

and constitute the major source of experimental error. We use a linear fitting to calculate the maximum dispersion from zero in function of pressure. This error in the volume reading is then divided by the amount of sample and corresponds to the error of the adsorbed gas in sample per unit of mass.

Pore volume and pore size distribution

The total pore volume is derived from the amount of vapor adsorbed at a relative pressure close to unity, by assuming that the pores are then filled with liquid adsorbate and it is given by the relation

$$V_{pore} = n_{fill} / \rho_{liq}$$

where SPV is the specific pore volume, n_{fill} is the adsorbed amount of gas at the relative pressure P/Po= 0.9 and ρ_{liq} is the liquid density of the adsorbate. The pores which are not filled below the relative pressure 1 would have a negligible contribution to the total pore volume and the surface area of the sample, the average pore size can be estimated using the relation:

$$r = 2*V_{pore}/S$$

where the r is the average pore size, V_{pore} is the specific pore volume and S is the surface area. Under the assumption of a cylindrical pore geometry and a type H1 hysteresis, the average pore diameter can be estimated.

	BET specific area (m^2/g)	Pore volume (cc/g) (P/Po = 0.9)	Average pore diameter (nm)
Argon 87.3K	235	0.221	3.76
Nitrogen 77.36K	309	0.294	3.78

Table S1. Estimated parameters from BET experiments.

From the desorption isotherm of Ar and N_2 the pore size distribution was calculated using non-local density functional theory (NLDFT) with the software ASiQwin 3.01. For both gases, the pore size distribution is very similar and in good agreement with the average pore diameter estimated from the pore volume.

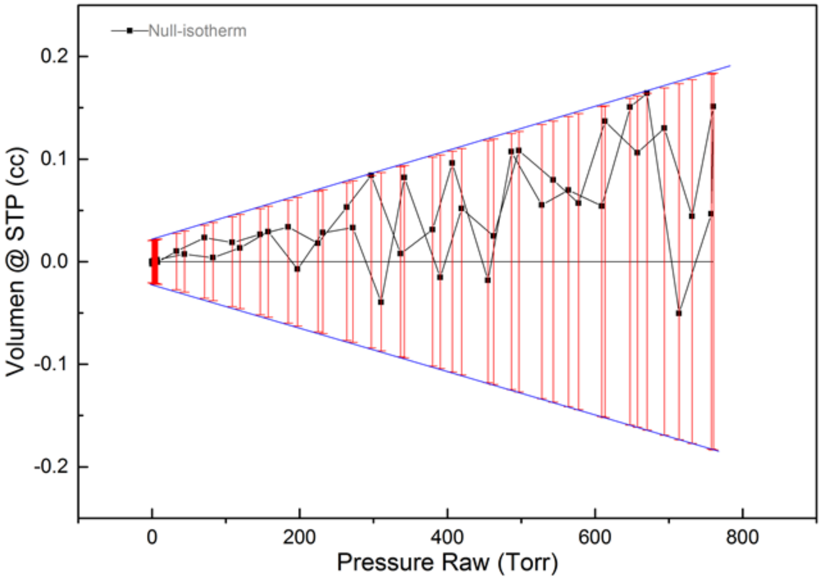

Figure S1. Empty cell experiment in units of volume (cc) STP, the experiment was made using an empty sample cell and then subtracting the calibration. The red bars mark the error related to the maximum variation of the recorded volume as a function of the pressure.

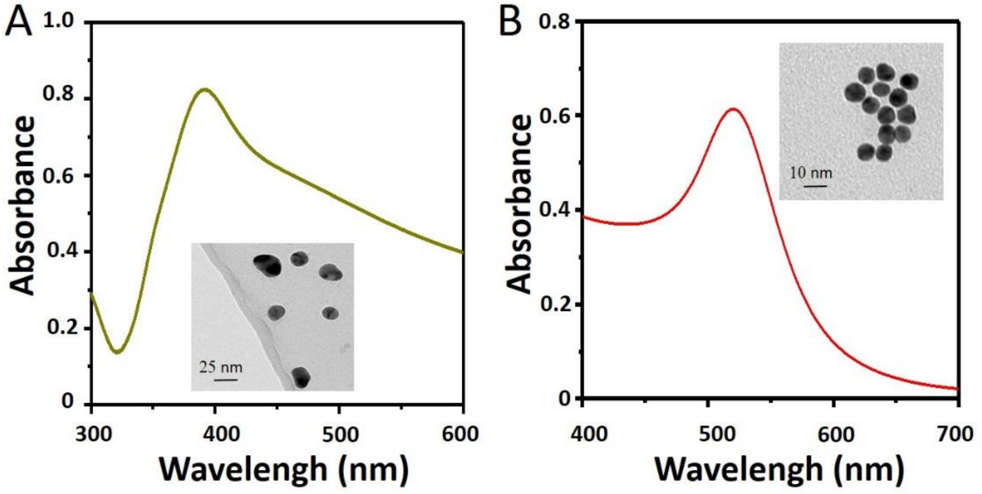

Figure S2. Characterization of nanoparticles colloidal solutions (A) UV spectrum of the colloidal solution of AgNPs (inset: TEM images corresponding to the colloidal AgNPs). (B) UV spectrum of the colloidal solution of AuNPs (inset: TEM images corresponding to the colloidal AuNPs).
